# Evaluation of the Effect of Concurrent Chronic Total Occlusion and Successful Staged Revascularization on Long-Term Mortality in Patients with ST-Elevation Myocardial Infarction

**DOI:** 10.1155/2014/756080

**Published:** 2014-02-10

**Authors:** Guoxiang Shi, Pengcheng He, Yuanhui Liu, Yaowang Lin, Xing Yang, Jiyuan Chen, Yingling Zhou, Ning Tan

**Affiliations:** Department of Cardiology, Guangdong Cardiovascular Institute, Guangdong General Hospital, and Guangdong Academy of Medical Sciences, Guangzhou 510080, China

## Abstract

*Aims*. To investigate the impact of chronic total occlusion (CTO) in non-infarct-related artery (IRA) on the long-term prognosis and evaluate the clinical significance of staged revascularization in patients with ST-segment elevation myocardial infarction (STEMI). *Methods*. 1266 STEMI patients with primary percutaneous coronary intervention (PCI) were categorized as single-vessel disease (SVD), multivessel disease (MVD) without and with CTO. We study the clinical outcomes of patients after primary PCI in the following 3 years. Additionally, patients with CTO received staged revascularization, and major adverse cardiac events (MACE) during 3-year follow-up were recorded. *Results*. Presence of CTO was a predictor of both early mortality [hazard ratio (HR) 3.4, 95% confidence interval (CI) 2.4–4.5, *P* < 0.01] and late mortality (HR 1.9, 95% CI 1.4–3.6, *P* < 0.01), whereas MVD without CTO was only a predictor of early mortality (HR 1.7, 95% CI 1.3–2.3, *P* < 0.05). In CTO group, 100 patients had successful CTO recanalization, and 48 patients failed. During 3-year follow-up, patients with failed procedure had higher cardiac mortality (22.9% versus 9.0%, *P* = 0.020) and lower MACE-free survival (50.0% versus 72.0%, *P* = 0.009) compared to patients with successful procedure. *Conclusion*. The presence of CTO and not MVD alone is associated with long-term mortality. Successful revascularization of CTO in the non-IRA is associated with improved clinical outcomes in patients with STEMI undergoing primary PCI.

## 1. Introduction

Acute ST-segment elevation myocardial infarction (STEMI) typically arises from sudden thrombotic occlusion of a coronary artery [1]. Treatment of patients with STEMI aims at early and sustained restoration of antegrade flow in the infarct-related artery (IRA). Timely and successful reperfusion leads to salvage of myocardium at risk and reduces mortality. Mechanical reperfusion by primary percutaneous coronary intervention (PCI) with stent implantation is currently the preferred treatment for patients presenting with STEMI. [2]. Approximately 40–65% of the STEMI patients have multivessel disease (MVD) and 10% to 13% of the patients have a chronic total occlusion (CTO) in a non-IRA [3–6]. However, there is only limited information about predictors of MVD or CTO in non-IRA in patients presenting with STEMI on long-term clinical outcome after primary PCI. Several studies have shown that successful percutaneous recanalization of CTO is associated with reduced long-term cardiac mortality [7–9]. However, it remains unclear whether successful staged recanalization of CTO in the non-IRA could improve clinical outcomes in patients with acute STEMI. The objective of the present investigation, given that the available data are limited, was to evaluate the effect of CTO in a non-IRA on the clinical outcomes in unselected patients presenting with STEMI. In addition, we examined the prognostic impact of complete percutaneous revascularization of CTO lesions in the non-IRA on long-term survival and occurrence of major adverse cardiac events (MACE) in patients with acute STEMI treated with primary PCI.

## 2. Materials and Methods

### 2.1. Study Population

From January 2005 to June 2009, a total of 1285 consecutive and unselected patients were admitted to our hospital with STEMI. Acute STEMI was diagnosed according to American Heart Association criteria including symptoms consistent with ongoing myocardial ischemia ≥30 min, accompanied by an electrocardiogram with ST-segment elevation ≥1 mm (0.1 mV) in two contiguous leads or more, new left bundle branch block, or true posterior infarction. Total 19 patients (1.5%) were lost to follow-up during the study, and remaining 1266 eligible patients (98.5%) constituted the study population.

### 2.2. Angiographic Analysis and PCI

All patients were given a loading dose of aspirin (300 mg) and clopidogrel (600 mg) immediately after arrival at emergency room and were then sent to catheterization laboratory and underwent immediate angiography with a view to perform primary PCI. If the coronary anatomy was suitable for PCI, the procedure was performed with standard techniques. Heparin (100 IU/kg) was administered before PCI. All procedural decisions, including device selection and adjunctive pharmacotherapy such as glycoprotein IIb/IIIa inhibitors, were made at the discretion of the operator.

Upon the operator's online assessment during emergency angiography, patients were categorized as having SVD, MVD without CTO, or MVD with concurrent CTO. For the purpose of this study, all angiograms were prospectively reviewed by two independent readers with discrepancies resolved by a third reader and consensus. MVD was defined as ≥1 stenosis >70% of the coronary lumen diameter in >1 of the non-infarct-related epicardial arteries or left main stenosis >50%. A CTO was defined as a total occlusion in a non-IRA before PCI without antegrade flow or with antegrade or retrograde filling through collateral vessels. Staged revascularization for these lesions was often performed at 7 to 10 days after primary PCI.

Complete revascularization was defined as a restoration of TIMI grade 3 flow with residual stenosis less than 30% on visual assessment in the three coronary arteries and their major branches (branch diameter >2 mm). Procedural success was defined as a final diameter stenosis <30% with a TIMI grade flow 3 of all the treated vessels without death, non-Q-wave or Q-wave myocardial infarction (MI), or emergency coronary surgery.

If a coronary stent was implanted, ticlopidine or clopidogrel was prescribed according to the guidelines, and aspirin (100 mg/day) was continued indefinitely. *β*-blockers, angiotensin converting enzyme inhibitors and statins were prescribed if not contraindicated.

### 2.3. Follow-Up

Data on baseline characteristics of study population, angiographic and procedural results during recanalization were prospectively collected and entered into a specific database. Data on clinical outcome were prospectively recorded and archived in the hospital's Electronic Patient Record System during the follow-up period. Follow-up information was obtained by direct telephone interviews and outpatient visits.

MACE including cardiac death, recurrent myocardial infarction, repeat revascularization (PCI and/or CABG), and rehospitalization because of heart failure were recorded.

### 2.4. Statistical Analysis

We performed a retrospective analysis of data which were prospectively collected according to the protocol of our institution. Outcomes were examined in groups of patients with SVD, MVD without a CTO, and MVD with a CTO in a non-IRA. Categorical variables are summarized as counts and percentages, while continuous variables with a normal distribution are reported as means ± standard deviation (SD). *χ* ^2^ test or Fisher's exact test were used for comparison of categorical variables, and Student's *t*-test was used to test differences among continuous variables. Cumulative event rates were estimated using Kaplan-Meier method, and difference in event rate between the groups was tested by log rank analysis. To assess the effect of particular parameters on mortality, multivariate analysis was performed using stepdown Cox proportional hazards regression modeling and expressed as the hazard ratio, with the 95% confidence interval. All clinical and angiographic variables were used in the risk-adjusted models. A value of *P* < 0.05 was considered statistically significant, and all *P* values are two-sided. All statistical analyses were performed using the software package SPSS, version 16 (SPSS Inc., Chicago, IL, USA).

## 3. Results

### 3.1. Patients and Procedures

During this study, total 19 patients (1.5%) lost follow-up, the remaining 1266 eligible patients (98.5%) completed with a follow-up duration of at least 3 years. Among the 1266 patients with STEMI, 595 patients (47%) had SVD, 519 patients (41%) had MVD without CTO, and 152 patients (12%) had MVD with a CTO lesion or more in the non-IRA. The baseline characteristics and angiographic characteristics of the study groups are listed in Table 1. Patients with MVD (with or without concurrent CTO) were older and more often had diabetes, hypertension, and hypercholesterolemia compared with SVD patients. In addition, the prevalence of cardiogenic shock on admission and previous myocardial infarction was greater in patients with CTO than that in patients with SVD and MVD without CTO. Less favourable baseline characteristics (had a lower left ventricular ejection fraction (LVEF), history of CHF, and renal insufficiency) were more common with greater severity of coronary artery disease. However, patients with MVD with or without a CTO were less often current smokers compared with SVD. Patients with a CTO in a non-IRA less often achieved postprocedural TIMI 3 flow in the IRA (SVD 88.9%, MVD without a CTO 82.9%, and MVD with a CTO 78.3%, *P* for trend = 0.001). During hospital stay, intra-aortic balloon pump (IABP) use was more frequent in patients with CTO than in patients with SVD and MVD without CTO. However, there was no difference of glycoprotein IIb/IIIa inhibitors (GPI) use between the three groups.

Among overall 152 patients with CTO in the non-IRA, one patient died during hospital stay before staged revascularization for CTO in the non-IRA, 100 patients (67.6%) received successfully staged revascularization (ranging 7–10 days) for CTO in the non-IRA, two patients with CTO and viable myocardium did not undergo PCI attempt because of patient or referring physician willingness for coronary surgery or medical therapy, 49 patients with CTO underwent PCI attempt but failure, and one of them received CABG and others refused that. Baseline clinical characteristics and angiographic features were well matched between the two groups (Table 2).

### 3.2. Clinical Outcomes

Overall 3-year, the rates of MACE and the individual endpoints of mortality increased significantly in patients with MVD (with or without concurrent CTO) compared with SVD patients. (Table 3). Meanwhile, during the overall 3-year follow-up duration, the rates of re-hospitalization due to heart failure, ischaemia-driven target vessel revascularization, and reinfarction were also higher in patients with MVD (with or without concurrent CTO) compared with SVD patients.

**Figure 1:**
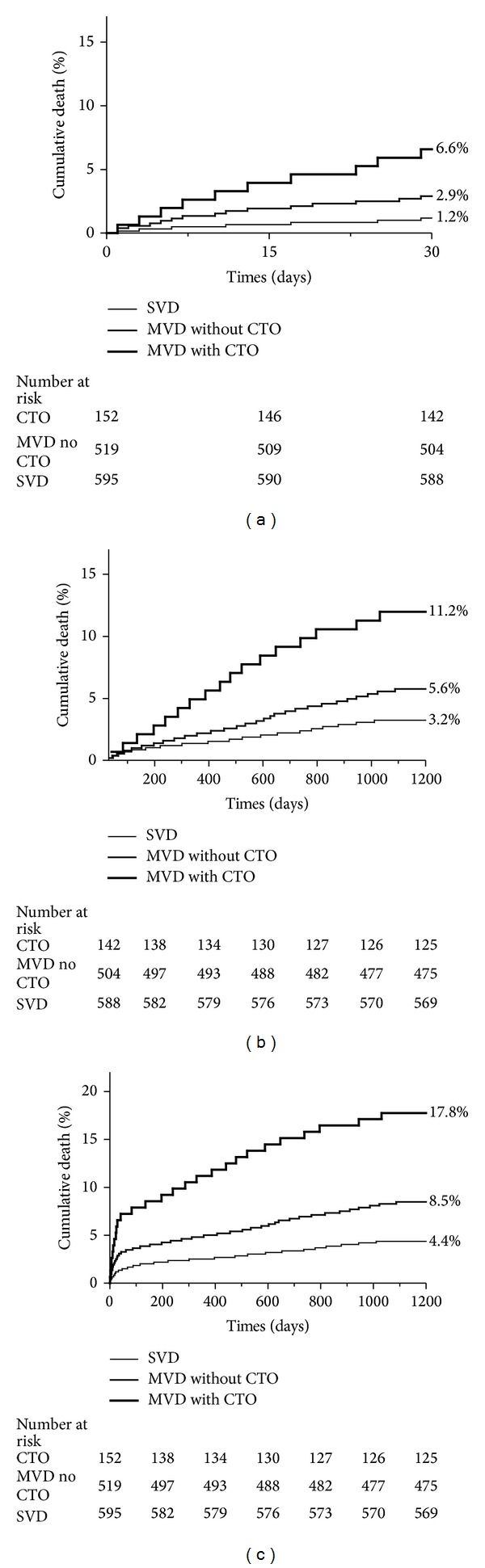
Time-to-event curves for mortality 0 to 30 days (a), mortality 30 days–3 years (b), and overall 3-year mortality (c) in patients with SVD, MVD without a CTO, and MVD with a CTO in a non-IRA. *P* values shown in figure are three-way. Pair-wise *P* values for 30-day mortality are CTO versus SVD, *P* < 0.001; MVD without a CTO versus SVD, *P* = 0.040; CTO versus MVD without a CTO, *P* = 0.035. Pair-wise *P* values for mortality between 30 days and 3 years are CTO versus SVD, *P* < 0.001; MVD without a CTO versus SVD, *P* = 0.043; CTO versus MVD without a CTO, *P* = 0.011. Pair-wise *P* values for overall 3-year mortality are CTO versus SVD, *P* < 0.001; MVD without a CTO versus SVD, *P* = 0.005; CTO versus MVD without a CTO, *P* = 0.001.

Figure 1 shows the cumulative mortality for patients with SVD, MVD without CTO, and MVD with a concurrent CTO during the first 30 days after STEMI and the 3 years thereafter. Between 0 and 30 days, Kaplan-Meier analysis revealed that mortality was significantly higher in patients with MVD concurrent CTO in a non-IRA (6.6%) than in those with MVD without a CTO (2.9%, *P* = 0.035) or SVD (1.2%, *P* < 0.001). Between 30 days and 3 years, the mortality curves continued to diverge, especially for patients with a non-IRA CTO. Mortality in 30-day survivors was significantly higher in patients with MVD concurrent CTO in a non-IRA (11.2%) compared with MVD without a CTO (5.6%, *P* = 0.011) and SVD (3.2%, *P* < 0.001). Furthermore, over the 3 year period, Kaplan-Meier analysis revealed that mortality was also significantly higher in patients with MVD concurrent CTO in a non-IRA (17.8%) than in those with MVD without a CTO (8.5%, *P* = 0.001) or SVD (4.4%, *P* < 0.001).

During 3-year follow-up, 23 (15.5%) patients with concurrent CTO died after discharge. In 48 patients with failed revascularization of a CTO in the non-IRA, 11 (22.9%) deaths were of cardiac origin caused by refractory heart failure (*n* = 7), acute myocardial infarction (*n* = 2), and fatal arrhythmic event (*n* = 2). Cardiac death occurred in 9 of 100 (9.0%) patients with successful recanalization of a CTO in the non-IRA due to refractory heart failure (*n* = 6), fatal arrhythmic event (*n* = 1), or acute myocardial infarction (*n* = 2). In additional, rehospitalization due to heart failure (22.9% versus 9.0%, *P* = 0.020) was more frequent in patients with failed recanalization of a CTO in the non-IRA. A trend was present towards more frequent repeat revascularization (25.0% versus 15.0%, *P* = 0.140) in patients with failed recanalization of a CTO in the non-IRA versus successful recanalization of a CTO in the non-IRA (Table 4).

**Figure 2:**
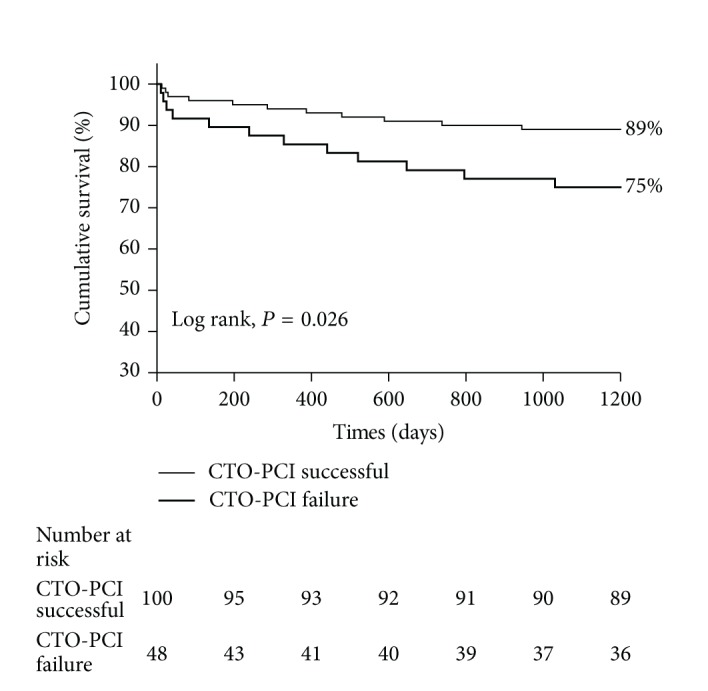
Cumulative survival at 3 years with respect to the outcome of attempted recanalization of a CTO in the non-IRA.

Figure 2 shows the cumulative survival for patients with failed revascularization of a CTO and successful recanalization of a CTO in the non-IRA during the 3 years. Kaplan-Meier analysis revealed that cumulative survival was higher (89.0% versus 75.0%, *P* = 0.026) in patients with successful revascularization of a CTO in the non-IRA than in those with failed procedure.

### 3.3. Predictors of Mortality

Table 5 shows independent multivariable predictors for death during the first 30 days, 30 days to 3 years and over 3 years, after primary PCI. The presence of a CTO in a non-IRA was found to be a strong and independent predictor for both 30-day mortality, with an HR of 3.4 (95% CI: 2.4 to 4.5, *P* < 0.01), and 30-day to 3-year mortality (HR: 1.9, 95% CI: 1.4 to 3.6, *P* < 0.01), while the presence of MVD without a concurrent CTO was also found to be a statistically significant independent predictor for 30-day mortality (HR: 1.7, 95% CI: 1.3 to 2.3, *P* < 0.05) but not for 3-year mortality excluding deaths within the first 30 days (HR: 1.1, 95% CI: 0.8 to 1.6, *P* = 0.51).

In multivariable Cox analysis of the patients with CTO, success revascularization of a CTO in the non-IRA was an independent predictor for both cardiac mortality (HR: 0.35, 95% CI: 0.19–0.68, *P* < 0.01) and MACE-free survival (HR: 0.58, 95% CI: 0.32–0.94, *P* < 0.01). Meanwhile, both LVEF and chronic renal insufficiency were associated with cardiac mortality and MACE-free survival, while previous myocardial infarction was associated with MACE-free survival but not with cardiac mortality (Table 6).

## 4. Discussion

In the present study, we sought to compare the early and long-term clinical results of patients with MVD who underwent primary PCI for STEMI with and without CTO in non-IRAs and evaluate the clinical significance of staged revascularization for a CTO in the non-IRA for patients with STEMI.

In this study involving consecutive and unselected patients presenting with acute STEMI, the principal findings from our investigation were as follows. First, the presence of MVD with a CTO in a non-IRA is associated with increased early mortality (within 30 days after STEMI), late mortality (from 30 days to 3 years after STEMI), and cumulative 3-year mortality. In contrast, MVD without a CTO was only associated with increased early mortality (within 30 days after STEMI). Second, successful staged revascularization of a CTO in the non-IRA was associated with an improved survival and reduced MACE in patients with acute STEMI treated with primary PCI.

The presence of MVD with a CTO has been associated with poorer clinical outcomes. The high mortality rate of STEMI patients with a concurrent CTO can in part be explained by patients with a CTO in a non-IRA had a higher prevalence of cardiovascular risk factors and comorbidities compared with SVD patients and MVD patients without a CTO [6, 10–13]. These patients tend to have lower left ventricular ejection fraction, lower baseline thrombolysis in myocardial infarction flow grades, history of diabetes and previous myocardial infarction, and cardiogenic shock on admission more often than patients without CTO. However, after adjustment for these differences in baseline characteristics, the presence of a CTO in a non-IRA remained a strong and independent predictor for early mortality and for late mortality. The presence of MVD without a CTO was only predictor for early mortality but not for late mortality.

Another explanation of the underlying mechanism for the increased mortality in patients with STEMI with concurrent CTO could be that in patients with CTO, they often are potentially at “double jeopardy” from the acute MI. As the distal coronary bed of the CTO largely depends upon collateral blood flow from the IRA, the area of risk of the IRA includes both its own supply territory and the myocardial distribution of the coronary artery in which the CTO is located, resulting in greater infarct size. The peak creatine phosphokinase and peak Troponin I levels, which were associated with infarct size. In previous trial [14, 15], which showed peak creatine phosphokinase and peak Troponin I levels tended to be higher in patients with a CTO than in patients without a CTO. Recently, Lexis et al. [16] have demonstrated that the presence of CTO in a non-IRA after STEMI is associated with worse reperfusion markers and larger enzymatic infarct size, both of which support this hypothesis.

Possible explanation of the underlying mechanism for the clinical benefit of CTO revascularization includes the following: first, improving the healing process of infarct border zone. Some myocardium located in infarct border zone changes from viable myocardium into stunning myocardium as result of the disruption of blood supply, and it is now widely believed that repetitive episodes of stunning (i.e., myocardial ischaemia) lead to development of myocardial apoptosis [17, 18]. With the restoration of myocardial blood supply, stunning myocardium will become viable. Second, recovering the contractile function of viable myocardium. Claessen et al. [19] reported that the presence of a CTO in a non-IRA in patients with STEMI is associated with reduced LVEF and further deterioration of LVEF, and Borgia et al. [17] have demonstrated that successful CTO PCI is associated with improved LVEF, with some studies finding that EF improvement especially stress EF (in response to dobutamine) following revascularization is associated with fewer cardiac events [20]. Both of them could translate into improvement in left ventricular function, slowdown of ventricular remodeling, decrease in electrical instability and associated risk of fatal arrhythmia, and increase in tolerance of future coronary occlusion events.

The presence of MVD without a CTO was only predictor for early mortality but not for late mortality. Goldstein et al. [11] have demonstrated the pathologic process in STEMI, shown that which involves not only the IRA but entire coronary tree, and can lead to the destabilization and rupture of multiple atherosclerotic plaques, resulting in a sharply increased risk repeated ischemic events of and death. Previous trials [21, 22] reported that the dynamics of this specific inflammatory process are the greatest in the first month after AMI, which possibly explains the increase in early mortality (within 30 days after STEMI), but not with late mortality in MVD without a CTO.

## 5. Study Limitations

Several limitations of the current study should be mentioned. *First*, this study is a retrospective observational study not a prospective randomized trial. *Second*, our study included patients from a single centre, and the number of patients was relatively small. *Third*, in many patients, the age of the CTO cannot be determined with confidence but was assumed after careful examination of the lesion morphology to be ≥3 month old and, consequently, considered as true CTO. *Forth*, the average follow-up period was relatively short; thus, more prolonged follow-up is needed to investigate whether such beneficial effects persist over time.

## 6. Conclusion

In patients with STEMI undergoing primary PCI, the poor prognosis of STEMI patients with MVD is driven by the presence of a CTO in a non-IRA. The presence of a CTO in a non-IRA is associated with both early and long-term mortality, even when early deaths are excluded from analysis, while MVD without a CTO is only associated with early mortality. In patients with acute STEMI treated with primary PCI, successful staged CTO percutaneous recanalization had a trend towards better cardiac survival and significant lower risk of MACE compared to patients with failed procedures during long-term follow-up.

## Figures and Tables

**Table 1:** Baseline clinical and angiographic characteristics.

Variable	SVD	MVD, no CTO	MVD with CTO	*P* value
	(*n* = 595, 47.0%)	(*n* = 519, 41.0%)	(*n* = 152, 12.0%)	
Baseline characteristics				
Age > 60 years	250 (42.0%)	222 (62.0%)	102 (67.1%)	<0.01
Male	410 (68.9%)	389 (75.0%)	121 (79.6%)	0.010
Diabetes mellitus	60 (10.1%)	98 (18.9%)	38 (25.0%)	<0.01
Hypertension	262 (44.0%)	296 (57.0%)	102 (67.1%)	<0.01
Hyperlipidaemia	238 (40.0%)	234 (45.1%)	85 (55.9%)	<0.01
Current smoking	310 (52.1%)	118 (42.0%)	66 (43.4%)	<0.01
Prior MI	46 (7.7%)	77 (14.8%)	47 (30.9%)	<0.01
Prior PCI	49 (8.2%)	82 (15.8%)	44 (28.9%)	<0.01
Cardiogenic shock on admission	42 (7.1%)	69 (13.3%)	36 (23.7%)	<0.01
CrCL (mL/min)	99 ± 37	84 ± 35	72 ± 34	<0.01
history of CHF	9 (1.5%)	17 (3.3%)	10 (6.6%)	<0.01
Family history of CVD (%)	250 (42.0%)	219 (42.2%)	58 (38.2%)	0.651
IABP, *n* (%)	24 (4.0%)	55 (10.6%)	36 (23.7%)	<0.001
Use of GPI, *n* (%)	301 (50.6%)	257 (49.5%)	76 (50.0%)	0.938
LVEF	52 ± 13	46 ± 14	36 ± 15	<0.01
LVEF < 40%, *n* (%)	125 (21.0%)	134 (25.8%)	72 (47.4%)	<0.001
Angiographic characteristics				
Single vessel disease	595 (100%)	0 (0%)	0 (0%)	—
2-vessel disease	0 (0%)	315 (60.7%)	74 (48.7%)	<0.001
3-vessel disease	0 (0%)	204 (39.3%)	78 (51.3%)	<0.001
Infarct-related artery				
LM, *n* (%)	1 (0.2%)	6 (1.2%)	3 (2.0%)	0.038
LAD, *n* (%)	253 (42.5%)	178 (34.3%)	67 (44.1%)	0.009
LCA, *n* (%)	72 (12.1%)	70 (13.5%)	30 (19.7%)	0.049
RCA, *n* (%)	269 (45.2%)	265 (51.0%)	52 (34.2%)	0.001
TIMI flow 3 post-PCI	529 (88.9.%)	430 (82.9%)	119 (78.3%)	0.001

Categorical variables are described as absolute numbers (%) and compared by means of the chi-square test; continuous variables are described as mean ± SD and compared by means of the Student's *t*-test. MI: myocardial infarction; PCI: percutaneous coronary intervention; CrCL: creatinine clearance; CHF: congestive heart failure; CVD: cardiovascular disease. LM: left main coronary artery; LAD: left anterior descending coronary artery; LCA: left circumflex artery; RCA: right coronary artery; TIMI: thrombolysis in myocardial infarction; PCI: percutaneous coronary intervention; IABP: intra-aortic balloon pump; GPI: glycoprotein IIb/IIIa inhibitors; LVEF: left ventricular ejection fraction.

**Table 2:** Baseline clinical and angiographic characteristics of patients with CTO who underwent PCI attempt.

Variable	CTO-PCI		*P* value
	Successful (*n* = 100, 68%)	Failed (*n* = 48, 32%)	
Baseline characteristics			
Age > 60 years	68 (68.0%)	32 (66.7%)	0.871
Male	78 (78.0%)	40 (83.3%)	0.450
Diabetes mellitus	23 (23.0%)	11 (22.9%)	0.991
Hypertension	65 (65.0%)	33 (68.8%)	0.652
Hyperlipidaemia	55 (55.0%)	28 (58.3%)	0.702
Current smoking	45 (45.0%)	19 (39.6%)	0.534
Prior MI	28 (28.0%)	16 (33.3%)	0.506
Prior PCI	28 (28.0%)	14 (29.2%)	0.883
Cardiogenic shock on admission	22 (22.0%)	10 (20.8%)	0.872
CrCL (mL/min)	73 ± 32	71 ± 33	0.727
Family history of CVD (%)	37 (37.0%)	19 (39.6%)	0.762
LVEF < 40%, *n* (%)	45 (45.0%)	23 (47.9%)	0.739
Angiographic characteristics			
Infarct-related artery			
LAD, *n* (%)	45 (45.0%)	21 (33.8%)	0.886
LCA, *n* (%)	20 (20.0%)	10 (20.8%)	0.906
RCA, *n* (%)	35 (35.0%)	17 (35.4%)	0.960
Number of diseased vessels			0.725
2	49 (49.0%)	25 (52.1%)	
3	51 (51.0%)	23 (47.9%)	
Non-IRA-CTO vessel			
LAD, *n* (%)	36 (36.0%)	20 (41.6%)	0.280
LCX, *n* (%)	30 (30.0%)	14 (29.2%)	0.917
RCA, *n* (%)	34 (34.0%)	14 (29.2%)	0.557
IABP, *n* (%)	21 (21.0%)	11 (22.9%)	0.791
CTO lesion length (mm)	23.4 ± 8.3	24.2 ± 7.1	0.544
Reference diameter (mm)	2.97 ± 0.38	2.92 ± 0.39	0.462
Number of stent per lesion	1.7 ± 0.6	—	—
Stented segment length (mm)	41.9 ± 17.6	—	—
Complete revascularization	84 (84.0%)	6 (12.5%)	<0.001
Total fluoroscopy time (min)	26.44 ± 7.1	25.53 ± 6.9	0.364
Time of staged procedure after primary PCI (day)	8.21 ± 1.23	8.17 ± 1.55	0.876

Categorical variables are described as absolute numbers (%) and compared by means of the chi-square test; continuous variables are described as mean ± SD and compared by means of the Student's *t*-test. MI: myocardial infarction; PCI: percutaneous coronary intervention; CrCL: creatinine clearance; CVD: cardiovascular disease. LAD: left anterior descending coronary artery; LCA: left circumflex artery; RCA: right coronary artery; LVEF: left ventricular ejection fraction; IABP: intra-aortic balloon pump; CTO: chronic total occlusion; PCI: percutaneous coronary intervention.

**Table 3:** Overall 3-year clinical outcomes of patients who underwent primary percutaneous coronary intervention.

Variable	SVD	MVD, no CTO	MVD with CTO	*P* value
	(*n* = 595, 47.0%)	(*n* = 519, 41.0%)	(*n* = 152, 12.0%)	
MACE	108 (18.2%)	131 (25.2%)	56 (36.8%)	<0.001
Death	26 (4.4%)	44 (8.5%)	27 (17.8%)	<0.001
Cardiac	15 (2.5%)	30 (5.8%)	24 (15.8%)	<0.001
Noncardiac	11 (1.9%)	14 (2.7%)	3 (2.0%)	0.470
Reinfarction	26 (4.4%)	42 (8.1%)	15 (9.9%)	0.009
Ischaemia-driven target vessel revascularization	63 (10.6%)	71 (13.7%)	27 (17.8%)	0.042
Rehospitalization due to heart failure	25 (4.2%)	43 (8.3%)	20 (13.2%)	<0.001
Stroke	9 (1.5%)	10 (1.9%)	5 (3.3%)	0.357

Categorical variables are described as absolute numbers (%) and compared by means of the chi-square test. CTO: chronic total occlusion; PCI: percutaneous coronary intervention; MACE: major adverse cardiac events.

**Table 4:** Overall 3-year clinical outcomes in patients with CTO of attempted recanalization of a CTO in the non-IRA.

Variable	CTO-PCI		*P* value
	Successful (*n* = 100, 67.6%)	Failed (*n* = 48, 32.4%)	
MACE	28 (28.0%)	24 (50.0%)	0.009
Death	11 (11.0%)	12 (25.0%)	0.028
Cardiac	9 (9.0%)	11 (22.9 %)	0.020
Noncardiac	2 (2.0%)	1 (2.1%)	0.973
Ischaemia-driven target vessel			
Revascularization	15 (15.0%)	12 (25.0%)	0.140
Rehospitalization due to			
heart failure	9 (9.0%)	11 (22.9%)	0.020
Stroke	3 (3.0%)	2 (4.1%)	0.713
Reinfarction	9 (9.0%)	6 (12.5%)	0.509

Categorical variables are described as absolute numbers (%) and compared by means of the chi-square test. CTO: chronic total occlusion; PCI: percutaneous coronary intervention; MACE: major adverse cardiac events.

**Table 5:** Independent predictors of early (0–30 days) and late (30 days–3 years) mortality.

Variable	HR (95% CI)	*P* value
Predictors of overall 3-year mortality		
Shock on admission	4.6 (3.5–6.7)	<0.01
MVD with CTO	2.3 (1.5–2.8)	<0.01
CrCL < 60 mL/min	1.6 (1.1–2.2)	<0.05
History of prior MI	1.6 (1.1–2.3)	<0.05
History of congestive heart failure	2.8 (1.7–4.7)	<0.01
LAD-related MI	2.1 (1.6–2.8)	<0.01
TIMI flow 3 post-PCI	0.6 (0.4–0.8)	<0.01
Predictors of 30-day–3-year mortality		
Shock on admission	2.1 (1.2–2.9)	<0.05
MVD with CTO	1.9 (1.4–3.6)	<0.01
MVD without CTO	1.1 (0.8–1.6)	0.50
History of congestive heart failure	2.6 (1.4–4.5)	<0.01
LAD-related MI	1.9 (1.5–2.4)	<0.01
Predictors of 30-day mortality		
Shock on admission	7.8 (5.9–9.8)	<0.01
MVD with CTO	3.4 (2.4–4.5)	<0.01
MVD without CTO	1.7 (1.3–2.3)	<0.05
LAD-related MI	1.5 (1.2–1.8)	<0.05
TIMI flow 3 after PCI	0.5 (0.3–0.8)	<0.01

CI: confidence interval; HR: hazard ratio; MVD: multivessel disease; CTO: chronic total occlusion; CrCL: creatinine clearance; LAD: left anterior descending coronary artery; MI: myocardial infarction; TIMI: thrombolysis in myocardial infarction; PCI: percutaneous coronary intervention.

**Table 6:** Multivariate analyses of predictors for cardiac mortality and MACE-free survival of patients with CTO.

Variable	HR (95% CI)	*P* value
MACE		
CTO-PCI successful	0.58 (0.32–0.94)	<0.01
Prior MI	3.89 (1.95–7.84)	<0.05
CKD	3.51 (1.52–7.87)	<0.01
LVEF (per-10% increment)	0.94 (0.88–0.99)	<0.05
Mortality		
CTO-PCI successful	0.35 (0.19–0.68)	<0.01
Age	1.08 (1.02–1.11)	<0.01
Prior MI	2.31 (0.73–7.35)	0.16
CKD	7.18 (2.95–17.40)	<0.01
LVEF (per-10% increment)	0.87 (0.77–0.93)	<0.01

CI: confidence interval; HR: hazard ratio; CTO: chronic total occlusion; PCI: percutaneous coronary intervention; MI: myocardial infarction; CKD: chronic kidney disease; LVEF: left ventricular ejection fraction.
